# Supplementary material for: MetaRibo-Seq measures translation in microbiomes
Source: Nat Commun. 2020 Jun 29;11:3268. doi: 10.1038/s41467-020-17081-z (PMC7324362; doi:10.1038/s41467-020-17081-z)
Supplement: Supplementary file 10 — Supplementary Data 7 [file 41467_2020_17081_MOESM10_ESM.zip › File2/Confidence_High_Taxonomy/107682_out.krona.html]

Javascript must be enabled to view this page.

members
magnitude
magnitudeUnassigned
count
unassigned
taxon
rank

107682\_out

95

2
superkingdom
95

976
phylum
95

200643
class
95

order
171549
95

1
171550
family

239759
genus
1

328814

SRS016381\_contig\_number\_2901
species
1

815
family
94


SRS011084\_contig\_number\_contig-100\_6942.79274SRS013951\_contig\_number\_41167SRS015579\_contig\_number\_contig-100\_7594.113530SRS019161\_contig\_number\_34950SRS043667\_contig\_number\_4384SRS053649\_contig\_number\_contig-100\_2500.2500SRS054059\_contig\_number\_contig-100\_14475.14476SRS064757\_contig\_number\_contig-100\_2677.144526SRS077024\_contig\_number\_5688SRS1041032\_contig\_number\_11395SRS1041137\_contig\_number\_contig-100\_19819.104410SRS1054691\_contig\_number\_contig-100\_14123.52156SRS1055067\_contig\_number\_16968SRS893292\_contig\_number\_5105SRS893295\_contig\_number\_5176
816
genus
94
15

species
2292935

SRS017191\_contig\_number\_contig-100\_788.121177SRS022137\_contig\_number\_contig-100\_486.135765SRS043701\_contig\_number\_11044SRS055982\_contig\_number\_contig-100\_900.113752SRS064232\_contig\_number\_2521SRS075963\_contig\_number\_contig-100\_1587.1587SRS101433\_contig\_number\_contig-100\_935.101945SRS893342\_contig\_number\_8805
8

1
2292956

SRS064276\_contig\_number\_27786
species


SRS023346\_contig\_number\_1717SRS147653\_contig\_number\_7143
817
species
2

1
2292943

SRS1041136\_contig\_number\_24496
species

2
species
246787

SRS024331\_contig\_number\_24248SRS048981\_contig\_number\_307

1
species
2292919

SRS017103\_contig\_number\_18191


SRS046717\_contig\_number\_2251
46506
species
1

2
2292944

SRS045195\_contig\_number\_3445SRS148874\_contig\_number\_2611
species

species

SRS013215\_contig\_number\_42SRS014613\_contig\_number\_4369SRS016018\_contig\_number\_1079
818
3

5
species

SRS019286\_contig\_number\_5196SRS057717\_contig\_number\_5680SRS1055043\_contig\_number\_27545SRS140645\_contig\_number\_558SRS147139\_contig\_number\_39994
671267

1

SRS020394\_contig\_number\_8912
1841855
species

species

SRS013476\_contig\_number\_21775SRS023715\_contig\_number\_12955SRS024132\_contig\_number\_37746SRS104636\_contig\_number\_124SRS144135\_contig\_number\_contig-100\_637.196381SRS146812\_contig\_number\_contig-100\_438.310779SRS893300\_contig\_number\_9623SRS971276\_contig\_number\_13331
2292917
8

2
28116

SRS043001\_contig\_number\_2243SRS048060\_contig\_number\_11182
species

species
2292939

SRS1055099\_contig\_number\_12896
1

37

SRS011134\_contig\_number\_contig-100\_1139.306976SRS012273\_contig\_number\_48339SRS013940\_contig\_number\_19220SRS015794\_contig\_number\_8249SRS015960\_contig\_number\_20688SRS017701\_contig\_number\_contig-100\_720.128933SRS017821\_contig\_number\_26591SRS018049\_contig\_number\_252SRS018575\_contig\_number\_4801SRS020328\_contig\_number\_contig-100\_3617.75537SRS022071\_contig\_number\_contig-100\_2009.222370SRS022524\_contig\_number\_14390SRS023583\_contig\_number\_15140SRS024549\_contig\_number\_4831SRS024663\_contig\_number\_contig-100\_147.98249SRS042628\_contig\_number\_35053SRS043768\_contig\_number\_28405SRS045713\_contig\_number\_1379SRS052078\_contig\_number\_1805SRS056259\_contig\_number\_contig-100\_781.167953SRS056519\_contig\_number\_8570SRS063040\_contig\_number\_425SRS063985\_contig\_number\_contig-100\_605.171104SRS075021\_contig\_number\_6062SRS077127\_contig\_number\_contig-100\_834.109015SRS078419\_contig\_number\_15401SRS098655\_contig\_number\_contig-100\_680.85901SRS098717\_contig\_number\_15862SRS101268\_contig\_number\_1067SRS1041039\_contig\_number\_contig-100\_250.161416SRS1041112\_contig\_number\_3274SRS1041145\_contig\_number\_28876SRS140513\_contig\_number\_9648SRS143598\_contig\_number\_contig-100\_5292.123275SRS145497\_contig\_number\_contig-100\_254.107282SRS148159\_contig\_number\_39054SRS148319\_contig\_number\_14390
357276
species

674529

SRS147557\_contig\_number\_contig-100\_14729.144998
species
1


SRS014923\_contig\_number\_45538SRS050925\_contig\_number\_contig-100\_757.184500SRS1041038\_contig\_number\_17287
457395
species
3
